# Characteristics of national registries for occupational diseases: international development and validation of an audit tool (ODIT)

**DOI:** 10.1186/1472-6963-9-194

**Published:** 2009-10-23

**Authors:** Dick Spreeuwers, Angela GEM de Boer, Jos HAM Verbeek, Frank JH van Dijk

**Affiliations:** 1 Academic Medical Center, University of Amsterdam, Department: Coronel Institute of Occupational Health, the Netherlands; 2 Finnish Institute of Occupational Health, Knowledge transfer team, Kuopio, Finland

## Abstract

**Background-:**

The aim of the study was to develop quality indicators that can be used for quality assessment of registries of occupational diseases in relation to preventive policy on a national level. The research questions were: 1. Which indicators determine the quality of national registries of occupational diseases with respect to their ability to provide appropriate information for preventive policy? 2. What are the criteria that can distinguish low quality from high quality?

**Methods-:**

First, we performed a literature search to assess which output of registries can be considered appropriate for preventive policy and to develop a set of preliminary indicators and criteria. Second, final indicators and criteria were assessed and their content validity was tested in a Delphi study, for which experts from the 25 EU Member States were invited.

**Results-:**

The literature search revealed two different types of information output to be appropriate for preventive policy: monitor and alert information. For the evaluation of the quality of the monitor and alert function we developed ten indicators and criteria. Sixteen of the twenty-five experts responded in the first round of the Delphi study, and eleven in the second round. Based on their comments, we assessed the final nine indicators: the completeness of the notification form, coverage of registration, guidelines or criteria for notification, education and training of reporting physicians, completeness of registration, statistical methods used, investigation of special cases, presentation of monitor information, and presentation of alert information. Except for the indicator "coverage of registration" for the alert function, all the indicators met the preset requirements of content validity.

**Conclusion-:**

We have developed quality indicators and criteria to evaluate registries for occupational diseases on the ability to provide appropriate information for preventive policy on a national level. Together, these indicators form a tool which can be used for quality improvement of registries of occupational diseases.

## Background

Exposure to occupational health risks accounts for a significant proportion of the burden of diseases [1,2] including a variety of social consequences [3], of which the estimated costs are considerable [4,5]. This burden could be substantially reduced through the application of proven risk-prevention strategies. Furthermore, new products, working practices and organisational contexts are continuously introduced into the working environment and bring with them new occupational diseases and work-related adverse health effects [6-8]. For these new emerging risks new risk-prevention strategies should be developed immediately.

Information about the incidence and distribution of occupational diseases is essential to develop these occupational health interventions for the purpose of prevention [9-12]. To enable companies, organisations of employers and employees, policy makers and occupational health professionals to set priorities for preventive policy and to evaluate interventions, information is needed about the severity and duration of diseases, and about their social and economic consequences. In the case of new emerging diseases, rapid detection of the health risks is necessary followed by an effective dissemination of knowledge to all stakeholders.

Most EU countries register occupational diseases in a national registry, while some have additional schemes for the surveillance of occupational diseases [13-15]. National registries are usually set up within the context of a financial compensation system for occupational diseases and are a part of the country's social security system. At the same time, such systems are intended to provide policy information for the prevention of occupational diseases. National registries are only one source, but mostly an authoritative one, of policy information. Various authors have recommended the use of a combination of monitoring systems and other data sources in order to assess working conditions, health effects and trends on a macro level as a more complete information input for preventive policy [16-19].

The registries of the various EU countries differ considerably regarding case definitions or diagnostic guidelines, criteria for notification or recognition, and the legal and social security context [20]. Furthermore, the level of under-reporting (as far as such is possible to define and assess) varies between countries [21]. Because of these differences, figures on occupational diseases are not comparable between European countries; moreover, the figures are often regarded as not reliable even within a country [22]. This calls for a more detailed study of the conditions that a registry has to meet in order to provide appropriate and reliable information for preventive policy.

According to Verma et al. (2002) prevention of occupational diseases can take place at the societal level and the workplace level [12]. The information need for these two levels is different. At the societal or national level, control measures are usually through regulatory actions and national policy. Information is needed on incidence of occupational diseases in sectors and occupations, the consequences and costs, as well as on new risks. At the workplace level information is needed on the nature of the hazard, where it is likely to be encountered, and the available options for risk control. In this study we focussed on information on a national level.

In line with Donabedian, we defined the quality of a registry as the extent to which it provides appropriate information for preventive policy [23]. The research questions were: 1. Which indicators determine the quality of registries for occupational diseases with respect to the ability to provide appropriate information for preventive policy on a national level? 2. What are the criteria that can distinguish high from low quality?

## Methods

We approached the research questions in two steps. We first performed a literature search to assess which output of registries are considered as appropriate for preventive policy and to develop a set of preliminary indicators and corresponding criteria. We then performed a Delphi study to assess the final content of the indicators and criteria and to test their content validity. A point of departure was the reporting of cases of occupational diseases by physicians.

### 1. Assessment of appropriate output and development of preliminary indicators and criteria

We performed a literature search in Medline through PubMed with three subsets of MeSH terms, combined with the Boolean term AND, and used the terms both as MeSH terms and text words. The first subset comprised the terms 'occupational diseases OR workplace'. The second comprised the terms 'registries OR notification OR mandatory reporting'. The third comprised the terms 'health policy OR prevention and control OR policy making OR public policy OR social control policy'.

We developed the preliminary indicators and corresponding criteria in an iterative process by discussing the information we had retrieved from the literature. As a starting point, we considered which information output of a registry would be appropriate for preventive policy. Next, we used the quality model of Donabedian to develop a model of stages and essential aspects in the process of registration [23]. This model has been used as a framework to develop a set of meaningful and comprehensive quality indicators related to the process. Finally, we discussed the criteria that would distinguish high from low quality for the various indicators.

### 2. Assessment of content validity

#### Participants

To assess the content validity of the indicators, we invited one expert from each of the then 25 EU countries (the study has been performed in 2005) to evaluate the quality of the indicators and corresponding criteria. The experts were selected either because they had published on registration of occupational diseases or because they had participated in international working groups on occupational diseases [24]. If an expert was not able to participate in the study, he or she was asked to suggest another expert in his or her own country.

#### Procedure

We used a modified Delphi technique to assess the content validity of the quality indicators [25-27]. The features of the Delphi method are anonymity, iteration and feedback [28]. The modified Delphi procedure we applied comprised two rounds. In the first round, we asked the experts if they agreed with our proposal concerning the appropriate output of a registry, and sought their opinion on the completeness of the set of indicators. We asked them to evaluate the relevance of the indicators for preventive policy (yes/no) and the corresponding criteria (good, too weak, too strong or not relevant). We invited them to suggest modifications and additions. In the second round, we asked the experts who had responded in the first round to comment on the adjustments we proposed based on their comments. We sent the questionnaires for the first round and a reminder in February respectively March 2005. The second-round questionnaires were sent in July 2005, the reminder in September 2005. Appendix 1 [see Additional file 1] and 2 [see Additional file 2] comprise a summarized version of the questionnaires of the first respectively the second round of the Delphi procedure.

#### Analysis

For each indicator we asked the experts if they considered it relevant to preventive policy. If more than 50% of the experts did not consider the indicator relevant, we proposed deleting or adjusting it. For each corresponding criterion, we asked the experts if they agreed with the criterion (good) or found it too weak, too strong or not relevant. If more than 50% of the experts did not agree with a criterion, we adjusted it. We discussed all the comments of the experts. If there were convincing arguments for making adjustments on the basis of their comments, we did so.

We calculated a content validity index (CVI) for the indicators by dividing the number of approvals by the total number of answers [24]. If more than 50% of the experts approved of the indicator in the first round, we used these results to calculate the CVI; if this was not the case, we used the results of the second round to calculate the CVI. In the literature, the acceptable level for the validity of indicators (in the sense of representing the quality of the registry) ranges from 0.70 to 0.80 [24]. We took 0.70 as the minimum level of the validity score. We followed the same procedure when calculating the percentage of experts that agreed with the corresponding criteria, or considered the criteria as too weak, too strong or not relevant.

We developed a sum score for both the monitoring function and the alert function of a registry with a maximum of ten points for each function, including weighted scores for each indicator. Finally we calculated a CVI for both sum scores.

## Results

### 1. Assessment of appropriate output and development of preliminary indicators and corresponding criteria

The literature search in Medline resulted in 184 articles and screening of the abstracts reduced this number to 44 relevant articles. We deduced from the literature two types of appropriate output of registries of occupational diseases within the scope of national preventive policy [13,15,29-33], namely alert information and monitor information. We called the ability of a registry to generate these types of information the alert function and the monitor function, respectively.

The purpose of the monitor function is to assess the nature, magnitude and distribution of already recognized occupational diseases over time, related to sectors of industries, occupational groups, gender and age categories. This information is essential in order to set priorities for preventive policy [11,13,15]. The monitoring of these characteristics over time is necessary, for example, to evaluate the effectiveness of preventive policy measures.

The purpose of the alert function is to discover new associations between new or existing occupational risk factors and diseases. The discovery of new or rare diseases, unusual patterns of already known or common diseases, and suspicious exposure-disease associations at the individual level can provide vital leads for a more conclusive scientific evaluation and verification. The information output of the system is signals for new and emerging risks. The discovery of the 'popcorn-worker's lung' is a recent example of the usefulness of an alert function and of the need to investigate signals [8,34,35]. A similar pattern of discovery can be shown for many occupational diseases.

Following Donabedian, we developed a model consisting of three stages in the process of registration of occupational diseases, namely structural preconditions, the process of diagnosis and notification, and the output of registries [23].

The model was used as a framework to develop a set of meaningful and comprehensive quality indicators related to the registration process. The control and decrease of occupational diseases as valuable potential outcomes of a registry could not be included in our analysis as they were considered to be largely dependent on the implementation of appropriate preventive measures which were outside the scope of this study.

Based on the literature, we proposed a preliminary set of ten indicators to determine the quality of the essential functions of a national registry. These indicators were:

A. Indicators of structural preconditions:

1. Completeness of notification form (with nine sub-items) [29,36,37]

2. Participation rate of physicians (the number of real reporters divided by the total number of potential reporters) [21]

3. Availability of criteria or guidelines for notification [38]

4. Education and training of reporting physicians [10,39]

B. Indicators of the process of diagnosis and notification:

5. Access of employees to reporting physicians [10,40]

6. Completeness of registration [21,40]

7. Statistical methods used [11,16,29,36]

8. Investigation of special cases [6-8]

C. Output indicators:

9. Presentation of monitor information (with five sub-items) [3-5,10,11,13,15,33]

10. Presentation of alert information [6-8,10,41].

Indicators 7 and 9 were considered relevant only to the monitor function, and indicators 8 and 10 only to the alert function. We formulated corresponding criteria for every indicator and sub-items both for the monitor and alert functions.

### 2. Assessment of content validity

In the first round of the Delphi study, sixteen (64%) of the twenty-five experts responded to the questionnaire. Experts that did not respond were from Austria, Finland, France, Germany, Greece, Latvia, Lithuania, Malta and Slovakia. In the second round, we sent the questionnaire with proposals for adjustment to the sixteen respondents; of these, eleven responded (69%). Table 1 shows the response in both rounds.

**Table 1:** Response to the Delphi study in both rounds from countries that responded one or two times.

**EU country**	**Response first round**	**Response second round**
Belgium	**x**	**x**
Cyprus	**x**	**x**
Czech Republic	**x**	**x**
Denmark	**x**	**x**
Estonia	**x**	**x**
Hungary	**x**	
Ireland	**x**	
Italy	**x**	**x**
Luxembourg	**x**	
Netherlands	**x**	**x**
Poland	**x**	
Portugal	**x**	
Slovenia	**x**	**x**
Spain	**x**	**x**
Sweden	**x**	**x**
United Kingdom	**x**	**x**
Total number of respondents	16/25	11/16

In general, most of the experts agreed with the proposed indicators and their corresponding criteria. Fourteen experts (88%) agreed with the proposed distinction between the alert and the monitor function. Two experts did not agree, but did not say why. One expert agreed with the distinction but stated that it would not be applicable in many national systems as they are mostly based on fixed lists of occupational diseases.

**Figure 1:**
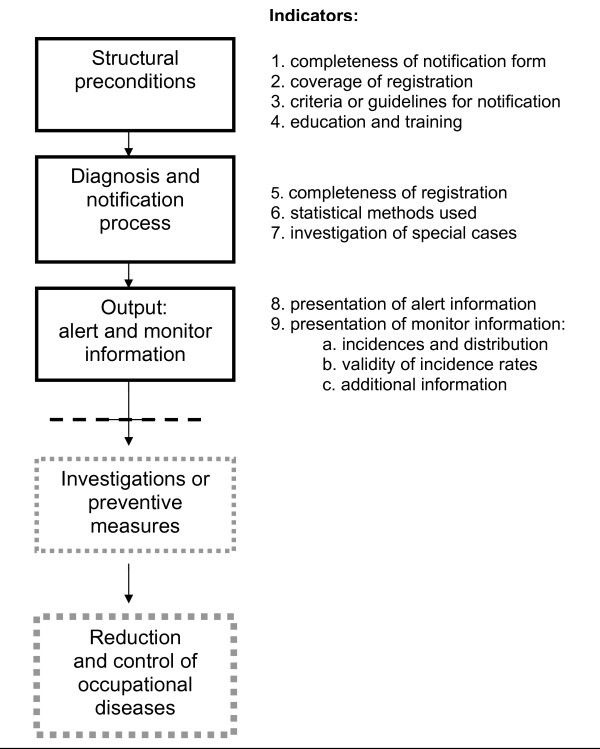
**Registry for occupational diseases and quality indicators: a model**.

Figure 1 presents the model of the process of registration for occupational diseases that we constituted on the basis of the quality model of Donabedian, and the final indicators after adjustments in the Delphi study. The preliminary set of ten indicators was considered complete by 79% of the experts. On the basis of the experts' comments, we omitted the indicator 'Participation of physicians', because it had considerable overlap with the indicator 'Completeness of Registration'. We renamed the indicator 'Access to notifying physicians' as 'Coverage of registration' and defined it as the proportion of the working population that has access to the consultancy hour of a physician who can report to the registry.

Appendix 3 presents the final indicators and the corresponding criteria after the adjustments resulting from the two rounds of the Delphi study [see Additional file 3]. The evaluation of the criteria by the experts is presented in the table as the proportion of experts who rated the criteria as good, too weak or too strong.

Concerning the indicator "completeness of the notification form" two experts stated that more information about exposure should be asked, such as the duration and intensity of exposure. One expert said that non-occupational factors should be taken into account. One expert stated that protective equipment and preventive actions should be registered, four experts said that the concept of 'susceptibility' was not feasible or should at least be clarified, and three experts said the same about the concept of 'probability of the causal relation'.

On the indicator "coverage of registration", one expert argued that coverage alone is not a good criterion: one may have nominal 90% coverage of poor quality, which is worse than having 45% coverage of better quality. Another aspect is appropriate information about the denominator, that is, about the working population covered. Furthermore, it is necessary for notifying physicians to cover the high-risk industries. One expert expressed a preference for a system in which both patient and employer can notify.

In the preliminary version we proposed as a criterion for the indicator 'criteria or guidelines for notification' that guidelines should be present for five reference diseases for the monitor function. On the basis of the experts' comments, we added mental health disorders as an additional reference disease. Because in the second round only two of the experts (18%) objected to this addition, we maintained this new reference disease.

In the preliminary version of the indicator, we proposed as a criterion for the indicator 'completeness of registration' that the participation level of notifying physicians should be more than 50% for both the alert function and the monitor function. On the basis of the experts' comments, we increased this level to 75%.

Concerning the presentation of incidence rates, a sub-item of the indicator 'presentation of monitor information', one expert stated that the denominator should not be the total workforce but the number of people exposed to the specific risk, because otherwise the incidence would be diluted. A comment on the sub-item "additional information" was that sickness absence is not relevant to diseases like hearing loss.

Table 2 shows the proposed calculation of a total quality score for the monitoring and the alert function. For the monitor function the content validity index for the calculation of the total score calculation was 0.55, for the alert function it was 0.70.

**Table 2:** Proposed calculation of a total quality score for the monitor and alert function of registries of occupational diseases

**Indicators Monitor function**	**Score**	**Indicators Alert function**	**Score**
Completeness of notification form	1 point	Completeness of the notification form	1 point
Coverage of registration	1 point	Coverage of registration	1 point
Guidelines or criteria for notification	1 point	Guidelines or criteria for notification	1 point
Education and training	1 point	Education and training	2 points
Completeness of registration	2 points	Completeness of registration	2 points
Statistical methods used	1 point	Investigation of special cases	2 points
Presentation of monitor information: - meeting the criteria for incidences - meeting the criteria for additional information - meeting the criteria for validity of incidences	(3 points) 1 point 1 point 1 point	Presentation of alert information	1 point
Max. score	10 points	Max. score	10 points

## Discussion

Both for the monitor function and the alert function we have assessed seven indicators, which determine the quality of registries of occupational diseases with respect to the ability to provide appropriate and reliable information for preventive policy on a national level. For every indicator we have assessed criteria that demarcate high and low quality. Except for the indicator "coverage of registration" for the alert function, all the indicators met the requirements of content validity. The calculation of the total score for the alert function met the requirements for content validity, whereas the calculation of the total score for the monitoring function did not. Together the indicators form a tool which we named "ODIT'. This tool can be used for quality assessment and quality improvement of registries of occupational diseases in relation to preventive policy.

A strong feature of our study is that we used a structured approach to develop the tool for quality assessment and improvement based on the system analysis of Donabedian. In addition, we were able to assess the content validity of the indicators by means of the Delphi technique.

An advantage of the tool is that it is easy to apply. It is possible to score the indicators with the aid of the annual report of the registry of a country and a concise questionnaire that can be sent to a limited number of key persons. In conclusion, the ability of a registry to provide information for preventive policy can be assessed and clues for quality improvement are provided. As far as we know, other tools for quality assessment and quality improvement of national registries of occupational diseases do not exist.

A limitation of the tool might be that we have reduced the case capturing and registration process to the straightforward notification of a case by a physician to the register. However in several registries employers and employees can also notify to the registry and validation of a case by a physician occurs in a later stage. Furthermore in several registries the case capturing phase can be divided in the notification of a case and the acknowledgement of a case, of which the latter is often related to criteria for financial compensation. As a result, some registries present two types of incidence rates, namely of reported cases and of acknowledged cases [42].

Another limitation of the tool is that it was difficult to formulate clear-cut criteria to demarcate high and low quality. For example, registries might have their own classifications of exposures or diseases, which might be more user friendly than the EU- shortlist of exposures [43] or the ICD-classification of diseases. Reporting physicians might prefer the classification of the registry, whereas using the EU- or ICD-classification would make the figures better comparable with figures from other registries. It should also be pointed out that the criteria to be met are minimum criteria: quality can still be further improved. For example, the criterion for the indicator "guidelines or notification criteria" requires the availability of guidelines for six reference diseases. But this criterion does not contain requirements for the underpinning scientific evidence of the guideline itself. Actually, there are many differences between the guidelines or criteria for notification of various countries. Moreover, the severity level of the disease in the criteria for notification might lead to over- or underreporting.

A further limitation is that although the tool does provide preconditions for reliable figures, considerable underreporting cannot be ruled out, even if the criteria of all the indicators would be fulfilled. Whether employees actually do visit a physician in case of an occupational disease and if physicians actually do report all cases of occupational diseases cannot be assessed with this tool.

Finally, the calculation of sum scores for the monitoring and alert function of registries might be criticized. One or more of the individual items might be more fundamental or even a prerequisite for the validity of the registry. Although we tried to account for this problem by using weighted scores, the sum score can only be used as a crude measure of the quality of a registry.

There are several complications, partly intrinsic to registries of occupational diseases, which has to be taken into account for the application of the tool. Because of the difference between the alert function and the monitor function, one monomorphous registry cannot be fully appropriate for both functions at the same time. For the alert function, it is desirable that as many physicians as possible participate in the system, as more alerts will be received from various industries and occupational groups. For the monitor function, it is more important that the group of reporting physicians, large or small, is rather stable, and that procedures do not change in a number of years, so that comparisons can be made over, for example, sectors of industry over time.

No single method of monitoring will be appropriate for all occupational diseases [18]. Registration is often no more than a matter of 'counting cases'. If cases can be confidently attributed to work in individual patients (as is the case with occupational injuries), counting cases will not be very complicated. But how does one proceed for diseases with recurrent episodes? How can one deal with diseases for which the relationship between a causal agent and a disease is difficult to assess, for example in the case of a reproductive hazard? Does disease monitoring suffice for prevention in the case of long latency? These and many other questions imply that different types of monitor instruments will be needed for different categories of diseases. Furthermore, information derived from other sources - such as epidemiological studies, and health and hazard surveys - will be necessary to provide additional information.

Incidence figures are composed on the basis of many individual reports of occupational diseases. But in many occupational diseases, occupational factors only account for the development of the disease in a part. Here, a better measure for preventive policy might be the excess of illness attributable to work [18]. We conclude that registration alone is not enough to provide all the figures needed. In addition, we should like to stress that the national monitoring of occupational diseases is a crude evaluation of preventive policy. In many cases, more detailed (sample) studies and assessments can provide better information. For example, in the case of noise-induced hearing loss monitoring may indicate that there is only a very slow decrease in the incidence after the start of a prevention programme. In addition to these finding, studies are needed to develop and evaluate effective ways to promote the use of hearing protectors or to implement interventions to decrease noise levels [44].

We conclude that the audit tool for the evaluation of the quality of a registry can support a process of quality improvement. Consequently, the costs of an improved register have to be weighted against the expected yields. A complication in the use of the tool for quality improvement might be the fact that in most countries national registries are based on compensation schemes, which can hamper the willingness to adjust the system for preventive purposes. The possible conflict of interests of physicians who notify and acknowledge occupational diseases might also play a role in the quality of the register. In some countries the physicians are employed by social of accident insurance companies and in other countries by hospitals or universities.

Concerning the measurement properties of the tool we developed, further research is needed on reproducibility and inter-observer variability.

The discussion with the experts on the indicators provided several issues for further research. Concerning the reporting physicians the differences in reporting behaviour, the possible causes of these differences and the effects of education and training on reporting behaviour are questions for further study. There is poor knowledge on what the barriers are that hinder employees to seek attention for an occupational disease and to turn to a physician, such as fear for adverse consequences. In regard to statistical methods the question how to determine the denominator is still in discussion. Using national figures of the working population might not be appropriate as the underreporting of cases is generally considered as large. The issue of which methods could be used to trace new and emerging risks, how to validate signals and, if there is sufficient evidence for assuming a novel cause, how to disseminate this information is an interesting field for further research.

## Conclusion

We have developed a valid tool for a quality assessment of registries of occupational diseases with respect to their ability to provide appropriate information for preventive policy on a national level, and called the tool "ODIT". The instrument can serve as a starting point for a quality improvement process. International collaboration should be fostered in order to improve and harmonize national registries.

## Key points

1. The output of registries for occupational diseases in relation to preventive policy can be divided in alert information and monitor information. Provision of the two types of information presupposes different requirements to a registry.

2. We have developed indicators for quality assessment of registries of occupational diseases related to preventive policy on a national level. Content validity of the indicators has been tested in a Delphi study. Together the indicators form an audit tool, which we have called "ODIT".

3. The ODIT can serve as a starting point for a quality improvement process of registries of occupational diseases.

## Competing interests

The authors declare that they have no competing interests.

## Authors' contributions

DS carried out the research and drafted the manuscript. AB has made substantial contributions to conception and design of the study and analysis and interpretation of data and revised the concept manuscript critically. JV has made substantial contributions to conception and design of the study and analysis and interpretation of data and revised the concept manuscript. FD has made substantial contributions to conception and design of the study and analysis and interpretation of data and revised the concept manuscript. All authors read and approved the final manuscript.

## Pre-publication history

The pre-publication history for this paper can be accessed here:



## Supplementary Material

Additional file 1 **Appendix 1: questionnaire first round**. Appendix 1 comprises a summarized version of the questionnaire of the first round of the Delphi procedure. Click here for file

Additional file 2 **Appendix 2: questionnaire second round**. Appendix 2 comprises a summarized version of the questionnaire of the second round of the Delphi procedure. Click here for file

Additional file 3 **Appendix 3: Quality indicators and criteria for national registries of occupational diseases**. Appendix 3 presents the final indicators and the corresponding criteria after the adjustments resulting from the two rounds of the Delphi study. Click here for file
