# Supplementary material for: Characteristics of national registries for occupational diseases: international development and validation of an audit tool (ODIT)
Source: BMC Health Serv Res. 2009 Oct 23;9:194. doi: 10.1186/1472-6963-9-194 (PMC2773237; doi:10.1186/1472-6963-9-194)
Supplement: Additional file 1 — Appendix 1: questionnaire first round. Appendix 1 comprises a summarized version of the questionnaire of the first round of the Delphi procedure. [file 1472-6963-9-194-S1.DOC]

Appendix 1: questionnaire first round

| Name:  Institute:  Country:  Date: |
| --- |
| 1. Do you consider the model for registries on occupational diseases, designed for quality evaluation, a sound model (see figure 1)?  2. Do you agree with the distinction in two functions, the monitoring function and the alert function, with respect to the ability to provide information for preventive policy?  3. Do you think the quality indicator set is complete? If not, which relevant indicators(s) is/are missing?  4-11: Do you think the following quality indicators are relevant for the monitoring function? Do you consider the criteria for good quality as too weak, good, too strong or not relevant?  4. “Completeness of notification form”?  5. “Participation of physicians”?  6. “Criteria or guidelines for notification”?  7. “Education and training”?  8. “Access to notifying physicians”?  9. “Completeness of registration”?  10. “Statistical methods used”?  11. “Monitor information”?  12. Do you agree that the quality indicator “investigation of special cases” is **not** relevant for the monitoring function?  13. With respect to the monitoring function we proposed that the indicators 1-7 can yield one point each, and indicator 10 can yield 3 points. The maximum quality score for the monitoring function will be 10. Do you agree with this proposed weighting of the indicators?  14-21. Do you think the quality indicators are relevant for the alert function? Do you consider the criteria for good quality as too weak, good, too strong or not relevant?  14. “Completeness of notification form”?  15. “Participation of physicians”?  16. “Criteria or guidelines for notification”?  17. “Education and training”?  18. “Access to notifying physicians”?  19. “Completeness of registration”?  20. “Investigation of special cases”?  21. “Alert information”?  22. Do you agree that the quality indicator “statistical methods used” is **not** relevant for the alert function?  23. With respect to the alert function we proposed that the indicators 1-6, 8 and 9 can yield one point each. The maximum quality score for the alert function will be 8. Do you agree with this proposed weighting of the indicators? |
