# Supplementary material for: Characteristics of national registries for occupational diseases: international development and validation of an audit tool (ODIT)
Source: BMC Health Serv Res. 2009 Oct 23;9:194. doi: 10.1186/1472-6963-9-194 (PMC2773237; doi:10.1186/1472-6963-9-194)
Supplement: Additional file 2 — Appendix 2: questionnaire second round. Appendix 2 comprises a summarized version of the questionnaire of the second round of the Delphi procedure. [file 1472-6963-9-194-S2.DOC]

Appendix 2: questionnaire second round

| Name:  Institute:  Country:  Date: |
| --- |
| **1. Indicator: “completeness of notification form” (sub-item susceptibility)**  a. A possibility is to skip this indicator-item, because it is difficult to assess for physicians. Would you agree to that?  b. We propose to adjust the criterion as follows:  “Information about susceptibility is a registered item AND susceptibility criteria are included in the six reference guidelines”.  Do you agree with our proposal? |
| **2. Indicator: “criteria or guidelines for notification”**  We propose to adjust the criterion as follows:  “Guidelines for assessment of occupational diseases on the European list are available. Guidelines contain at least requirements for diagnosis and exposure. This is evaluated in 6 reference diseases (occupational asthma, occupational hearing loss, contact dermatitis, asbestos related diseases, mental health disorders and musculoskeletal disorders of the upper limb)”.  Do you agree with our proposal? |
| **3. Indicators: “Participation of physicians and “Access to notifying physicians”**  We propose to replace these two indicators by one indicator “coverage of registration” for both the alert and the monitoring function.  As criterion we propose:  “Notifying physicians must cover at least 75% of the working population”.  Do you agree with our proposal? |
| **4. Indicator: “completeness of registration”**  We propose to adjust the criterion as follows:  Participation level> 75% of group of notifying physicians or of a sample of physicians with a known population, representative for the whole working population.  Do you agree with our proposal? |
| **5. Indicator: “monitor information”**  We propose to adjust the criterion as follows:  Information about sickness absence OR information about economic costs has been published for the six reference occupational diseases.  Do you agree with our proposal? |
| **6. Score of monitoring function:**  We propose the following adjusted score (max. score: 10 points):  Completeness of the notification form: 1 point  Coverage of registration 1 point  Guidelines or criteria for notification: 1 point  Education and training: 1 point  Completeness of registration: 2 points  Statistical methods used: 1 point  Monitoring information: 3 points  Do you agree with our proposal? |
| **7. Indicator: “completeness of registration”**  We propose to adjust the criterion for the alert function as follows:  Participation level> 75% of group of notifying physicians.  Do you agree with our proposal? |
| **8. Score of alert function:**  We propose the following adjusted score (max. score: 10 points):  Completeness of the notification form: 1 point  Coverage of registration 1 point  Guidelines or criteria for notification: 1 point  Education and training: 2 points  Completeness of registration: 2 points  Investigation of special cases: 2 points  Alert information: 1 point  Do you agree with our proposal? |
