# Supplementary material for: Characteristics of national registries for occupational diseases: international development and validation of an audit tool (ODIT)
Source: BMC Health Serv Res. 2009 Oct 23;9:194. doi: 10.1186/1472-6963-9-194 (PMC2773237; doi:10.1186/1472-6963-9-194)
Supplement: Additional file 3 — Appendix 3: Quality indicators and criteria for national registries of occupational diseases. Appendix 3 presents the final indicators and the corresponding criteria after the adjustments resulting from the two rounds of the Delphi study. [file 1472-6963-9-194-S3.DOC]

**Appendix 3: Quality indicators and criteria for national registries of occupational diseases**

| **Indicator:** | **Content Validity Index**  **Monitor**  **function** | **Criteria for Monitor function:** | **Evaluation of criteria,**  **% of experts/100** | **Content Validity Index**  **Alert function** | **Criteria for Alert function:** | **Evaluation of**  **criteria,**  **% of experts/100** |
| --- | --- | --- | --- | --- | --- | --- |
| ***Structural preconditions*** |  |  | Good Too Too  Weak Strong |  |  | Good Too Too  Weak Strong |
| ***1. Completeness of notification form:***  *a. diagnosis:*  *b. exposure:*  *c. occupation:*  *d. economic sector:*  *e. susceptibility:*  *f. probability of the causal relation:*  *g. age of worker:*  *h. sex of worker:*  *i. other causes:* | 0.94  1.00  1.00  1.00  0.88  0.88  0.94  1.00  0.85 | The notification form meets the criteria for diagnosis and exposure and at least for three other indicator items  Diagnosis according to the ICD-10 classification is a registered item  Type of exposure according to the EU shortlist [43] is a registered item Information about occupation is a registered item  Information about economic sector is a registered item  ^2)^  Probability of the causal relation is a registered item  Age is a registered item  Sex is a registered item  Other (additional) causes can be reported on the registration form | 0.79 0.21 0.00  0.94 0.06 0.00  **0.69** 0.25 0.06  0.75 0.25 0.00  0.71 0.21 0.07  0.79 0.21 0.00  1.00 0.00 0.00  1.00 0.00 0.00  0.91 0.00 0.09 | 0.92  1.00  1.00  1.00  0.88  0.79  0.93  1.00  0.92  0.75 | Six out of nine indicator items meet the proposed criteria  *and*  the notification form must offer the possibility to add relevant information  Diagnosis is a registered item  Type of exposure is a registered item  Information about occupation is a registered item  Information about economic sector is a registered item  Information about susceptibility is a registered item Probability of the causal relation is a registered item  Age is a registered item  Sex is a registered item  Other (additional) causes can be reported on the registration form | **0.58**  0.33 0.08  0.86 0.14 0.00  0.87 0.13 0.00  0.79 0.21 0.00  0.83 0.17 0.00  **0.54**  0.23 0.15  **0.62** 0.23 0.15  1.00 0.00 0.00  1.00 0.00 0.00  0.89 0.11 0.00 |
| ***2.*** ***Coverage of registration:****^3)^* | 0.80^1)^ | Notifying physicians must cover at least 75% of the working population | 0.80^4)^ | **0.67**^1)^ | Notifying physicians must cover at least 75% of the working population | **0.67**^4)^ |
| ***3. Guidelines or criteria for notification:*** | 1.00 | Guidelines for assessment of occupational diseases on the European list are available. Guidelines contain at least requirements for diagnosis and exposure. This is evaluated in six reference diseases (occupational asthma, occupational hearing loss, contact dermatitis, asbestos-related diseases, mental health disorders and musculoskeletal disorders of the upper limb) | 0.82^4)^ | 0.87 | The system must be open (that means, if there is a list it must be possible to report cases that are not on the list) | 0.85 0.15 0.00 |
| ***4.*** ***Education and training:*** | 0.94 | Diagnosis, guidelines for assessment and notification procedures are part of the medical specialist training or the postgraduate training for notifying physicians | **0.67** 0.33 0.00 | 0.93 | Diagnosis, guidelines for assessment and notification procedures are part of the medical specialist training or the postgraduate training for notifying physicians | 0.71 0.14 0.14 |
| ***Diagnosis and notification process*** |  |  |  |  |  |  |
| ***5. Completeness of registration:*** | 0.81 | Participation level  > 75 % of group of notifying physicians or of a sample of physicians with a known population, representative of the whole working population | 0.80^4)^ | 0.73 | Participation level  > 75 % of group of notifying physicians | 0.70^4)^ |
| ***6. Statistical methods used:*** | 0.79 | A public document that accounts for the statistical methods is available | 0.92 0.08 0.00 |  |  |  |
| ***7. investigation of special cases:*** |  |  |  | 0.86 | There are facilities to investigate special cases in the opinion of the representatives of the institute responsible for registration of occupational diseases | 0.72 0.14 0.14 |
| ***Output*** |  |  |  |  |  |  |
| ***8. Presentation of alert information:*** |  |  |  | 1.00 | Remarkable cases and clusters of cases must be mentioned in an annual report or in another publication | 0.79 0.07 0.14 |
| ***9. Presentation of monitor information:***  *a. Occupational diseases incidence rates and distribution:*  *a1. Incidence rates of specific occupational diseases for the total working population:*  *a2. Incidence-rates of specific occupational diseases by sector or by occupation:*  *a3. Distribution of occupational diseases by socio-demographic variables age and sex:*  *b. Additional information:*  *c. Validity of incidence rates:* | 0.93  0.93  0.87  0.73  0.85 | Incidence rates are presented for the six reference occupational diseases in the total working population  Incidence rates for the six reference occupational diseases by sector or by occupation are presented  Incidence rates for age and sex are given for the six reference occupational diseases  Information about sickness absence has been published for the six reference occupational diseases OR information about economic costs has been published for the six reference occupational diseases  Incidence rate of total occupational diseases must be at least 100/100,000 employees  *and*  Reports on the system must contain a consideration about the validity of the incidence rates for the six reference diseases | 0.71 0.21 0.07  0.73^4)^  0.73^4)^  0.73^4)^  0.73^4)^ |  |  |  |

1) Context validity score is derived from the second round

2) Susceptibility was omitted as an indicator item after the second round

3) The appointment of this indicator was the result of an adjustment based on comments made by the experts

4) Evaluation of criteria is derived from the second round; we asked if they could agree with the adjusted criteria

(Note: The numbers in the column “evaluation of criteria“ do not always add up to 1.00 because of rounding off)
